# Artemisinin Mediates Its Tumor-Suppressive Activity in Hepatocellular Carcinoma Through Targeted Inhibition of FoxM1

**DOI:** 10.3389/fonc.2021.751271

**Published:** 2021-11-24

**Authors:** Deeptashree Nandi, Pradeep Singh Cheema, Aakriti Singal, Hina Bharti, Alo Nag

**Affiliations:** Department of Biochemistry, University of Delhi, New Delhi, India

**Keywords:** artemisinin (ART), FOXM1 (Forkhead box M1), hepatocellular carcinoma (HCC), anticancer, anticarcinogenic agent, drug resistance

## Abstract

The aberrant up-regulation of the oncogenic transcription factor Forkhead box M1 (FoxM1) is associated with tumor development, progression and metastasis in a myriad of carcinomas, thus establishing it as an attractive target for anticancer drug development. FoxM1 overexpression in hepatocellular carcinoma is reflective of tumor aggressiveness and recurrence, poor prognosis and low survival in patients. In our study, we have identified the antimalarial natural product, Artemisinin, to efficiently curb FoxM1 expression and activity in hepatic cancer cells, thereby exhibiting potential anticancer efficacy. Here, we demonstrated that Artemisinin considerably mitigates FoxM1 transcriptional activity by disrupting its interaction with the promoter region of its downstream targets, thereby suppressing the expression of numerous oncogenic drivers. Augmented level of FoxM1 is implicated in drug resistance of cancer cells, including hepatic tumor cells. Notably, FoxM1 overexpression rendered HCC cells poorly responsive to Artemisinin-mediated cytotoxicity while FoxM1 depletion in resistant liver cancer cells sensitized them to Artemisinin treatment, manifested in lower proliferative and growth index, drop in invasive potential and repressed expression of EMT markers with a concomitantly increased apoptosis. Moreover, Artemisinin, when used in combination with Thiostrepton, an established FoxM1 inhibitor, markedly reduced anchorage-independent growth and displayed more pronounced death in liver cancer cells. We found this effect to be evident even in the resistant HCC cells, thereby putting forth a novel combination therapy for resistant cancer patients. Altogether, our findings provide insight into the pivotal involvement of FoxM1 in the tumor suppressive activities of Artemisinin and shed light on the potential application of Artemisinin for improved therapeutic response, especially in resistant hepatic malignancies. Considering that Artemisinin compounds are in current clinical use with favorable safety profiles, the results from our study will potentiate its utility in juxtaposition with established FoxM1 inhibitors, promoting maximal therapeutic efficacy with minimal adverse effects in liver cancer patients.

## Introduction

Hepatocellular carcinoma (HCC) is the predominant malignancy of the liver, being the sixth most common carcinoma and the fourth leading cause of cancer-related deaths worldwide (1). Intrahepatic metastasis renders surgical intervention largely ineffective and the five year survival rate of HCC patients following surgery is persistently low, owing to late diagnosis and lack of efficient therapy in addition to the highly aggressive nature of this cancer (2–4). Current treatment strategies such as liver transplantation, radiotherapy and chemotherapy come with their inadvertent drawbacks, including 50% possibility of tumor recurrence and poor efficacy of systemic pharmacotherapy (5). The only two drugs for the management of advanced HCC are sorafenib and regorafenib, characterized by low durable response rate and a median increase in survival by merely two to three months (6, 7). These are small molecule inhibitors and both these multikinase inhibitors act by suppression of various cellular kinases that participate in tumorigenic growth- while sorafenib blocks Raf, platelet derived growth factor receptor (PDGFR) and vascular endothelial growth factor receptor (VEGFR), regorafenib acts through hindering VEGFR1-3, PDGFRβ, fibroblast growth factor receptor 1, TIE2, c-KIT, RET and Raf (2, 7). Furthermore, the failure of these drugs to differentiate tumor cells from their adjacent non-tumor counterparts insinuates severe side effects such as weight loss, diarrhea, severe hypertension, myocardial infarction and gastrointestinal bleeding. Thus, management and therapy of HCC poses a challenge for the physicians as is evident from the surge in HCC incidence over the past decade. This evokes a dire need of research initiatives for comprehensive understanding of the fundamental molecular mechanisms triggering HCC and design of alternative anticancer strategies to circumvent contemporary limitations.

Accumulating evidence has shown paramount interest in natural products with potent chemotherapeutic and chemopreventive activities and minimal side effects. Accordingly, phytochemicals are being exploited for their optimal use in effective cancer treatment, including HCC (8, 9). Artemisinin, isolated from the traditional Chinese medicinal sweet wormwood plant (*Artemesia annua L*), has been the first line of defense against malaria, especially in its resistant forms (10–12). Recent reports have exhibited Artemisinin to possess dynamic anticancer properties against carcinoma of the gastric, colon, cervix and breast (13–18). Its basic mode of anti-carcinogenic action, cleavage of its endoperoxide bridge by free iron to release free radicals that induce cytotoxicity, is similar to its antimalarial function (19). The foremost incentive for developing Artemisinin as an anticancer agent is attributed to its low toxicity and high selectivity for cancer cells. Cancer cells display an insatiable appetite for iron uptake to facilitate unhindered proliferation. The large amount of cell surface transferrin receptors on tumor cells allows preferential uptake of Artemisinin relative to normal cells (20). Artemisinin and its derivatives have been studied in HCC models (21) and are claimed to exert cytotoxicity in HCC primarily by inducing apoptosis through modulation of cell cycle regulators like cyclin D1 and E, cyclin-dependent kinase 2 and 4, E2F1, p21, p27 and apoptotic drivers such as caspase 3, Bax and Bcl2 (22, 23). Nevertheless, detailed investigation about the molecular targets of Artemisinin in tumor cells remains elusive, thus restraining its translation in clinical settings and limiting its therapeutic efficacy.

FoxM1, a member of the forkhead box family of evolutionarily conserved transcription factors, is well- known for its imperative role in various physiological functions, including cell cycle regulation, DNA damage repair and apoptosis and is ubiquitously expressed in proliferating and regenerating mammalian cells (24, 25). FoxM1 was recently recognized to be deregulated during initiation and progression of multiple carcinomas, including malignancies of the prostate (26, 27), lungs (28), breast (29), pancreas (30), glioma (31) and liver (32). FoxM1 overexpression has been linked to major hallmarks of cancer, such as cellular hyper-proliferation, genomic instability, angiogenesis, metastasis and suppressed senescence (33–35). Its indispensible role in carcinogenesis and contribution to chemoresistance and radioresistance in various cancer types (35–38) makes it a prospective target for development of promising anticancer therapeutics. FoxM1 promotes proliferative expansion during HCC development (39–41), with several lines of evidences claiming up-regulated FoxM1 as a marker for aggressive HCC and poor prognosis (42, 43). FoxM1 inactivation, therefore, leads to inhibition of HCC progression and invasion (32, 44, 45). Moreover, HCC patients with lower level of FoxM1 were found to respond better to chemotherapy, implying the importance of targeting this oncogene for improved efficacy of anticancer strategies. In agreement, sorafenib was demonstrated to induce p53-mediated apoptosis and tumor suppression in HCC by targeted inhibition of FoxM1 (46).

Interestingly, a recent study in head and neck carcinoma hinted towards the possible role of FoxM1 in cell cycle arrest induced by dihydroartemisinin, an Artemisinin derivative (47). However, detailed research revolving around FoxM1’s mechanistic contribution in Artemisinin-driven therapy of hepatocarcinogenesis is still lacking. Our findings have identified FoxM1 as an important inhibitory target of Artemisinin in hepatic cancer cells. We showed that Artemisinin transcriptionally repressed FoxM1 and its downstream targets by intervening with its *trans*-activation ability in HCC cells, thereby blocking the deregulated expression of its pro-oncogenic downstream molecules. Importantly, we demonstrated FoxM1 knockdown to sensitize resistant HCC cells to Artemisinin therapy, signifying a novel mode of treatment for resistant HCC cases. Finally, our study depicted Artemisinin to act in an additive manner with Thiostrepton, a known FoxM1 inhibitor, to effectively stifle hepatic tumorigenesis. Collectively, our results established FoxM1 as an indispensible molecular target of Artemisinin in HCC and verified the potential of repurposing Artemisinin, preferably in combination with a FoxM1 inhibitor, as an innovative anticancer therapy for control of HCC.

## Materials and Methods

### Drugs, Antibodies and Plasmid Constructs

Artemisinin (Cat #361593) was purchased from Sigma-Aldrich and dissolved in pure ethanol for use. Monoclonal antibody against Flag (Cat #F3165) was procured from Sigma-Aldrich (Sigma, St. Louis, MO). Monoclonal antibodies for Aurora B Kinase (Cat #365200, RRID : AB_2533266) and Skp2 (Cat #323300, RRID : AB_2533074) were purchased from Invitrogen (Carlsbad, CA) while antibodies specific for FoxM1 (Cat #sc-500, RRID : AB_631521), Plk1 (Cat #sc-17783, RRID : AB_628157), CyclinB1 (Cat #sc-245, RRID : AB_627338), Survivin (Cat #sc-17779, RRID : AB_628302), PARP (Cat #sc-8007, RRID : AB_628105) and GAPDH (Cat #sc-32233, RRID : AB_627679) were procured from Santa Cruz Biotechnology Inc. (Santa Cruz, CA). Antibodies against E-cadherin (Cat #3195, RRID : AB_2291471), N-cadherin (Cat #13116, RRID : AB_2687616), Vimentin (Cat #5741, RRID : AB_10695459), ZEB-1 (Cat #3396, RRID : AB_1904164), Snail (Cat #3879, RRID : AB_2255011), Slug (Cat #9585, RRID : AB_2239535), cleaved caspase 9 (Cat #9505, RRID : AB_2290727) and cleaved caspase 7 (Cat #9491, RRID : AB_2068144) were obtained from Cell Signaling Technology (Danvers, MA).

The Flag-FoxM1 expression plasmid was generated by in-frame cloning of PCR amplified Flag-FoxM1 in pcDNA3.1 mammalian expression vector, using HindIII and XhoI restriction sites, as described previously (48). Transfection in HepG2 cells was carried out using FuGENE transfection reagent (Roche Diagnostics, Indianapolis, USA) according to manufacturer’s instructions.

### Cell Culture and Western Blotting

Mammalian Phoenix Ampho, HEK293T cells and hepatocellular carcinoma cell lines HepG2 and Hep3B were cultured in DMEM (Invitrogen, Carlsbad, CA), supplemented with 10% fetal bovine serum (Invitrogen, Carlsbad, CA), 100 U/ml of penicillin and 100 mg/ml streptomycin (Invitrogen, Carlsbad, CA) in a humidified incubator with 5% CO_2_ atmosphere at 37°C, as described earlier (48). The cell lines were purchased from the National Centre for Cell Science, provider of authenticated cell lines, who had conducted *Mycoplasma* contamination test by Hoechst staining and PCR. Cells with low passage numbers were used in this study.

Protein levels in the cells were detected by western blot method, performed as previously described (48). In brief, cells were harvested, washed and lysed with assistance of lysis buffer containing 50 mM Tris-HCl pH 7.5, 400 mM NaCl, 10% glycerol, 5 mM EDTA, 0.2% Nonidet P-40, 2 mM phenylmethanesulfonyl fluoride, 2 mM NaF, 1 mM Na_3_VO_4_ and protease inhibitor cocktail (Roche Applied Science, Mannheim, Germany). Protein concentration was estimated using Bradford’s reagent. Equal amount of protein lysates were subjected to SDS-PAGE followed by transfer onto polyvinylidene difluoride (PVDF) membrane (Millipore, Billerica, MA, USA). The membrane was blocked with 5% non-fat milk for 1 h, incubated with specific primary and horseradish peroxidase conjugated secondary antibodies and developed employing enhanced chemiluminescence.

### Generation of Stable Cell Line

The pSuper-Retro vector system was used for expression of shRNA in mammalian cells as described earlier (48). Recombinant retroviruses were produced in Phoenix Ampho packaging cell line. Hep3B cells with stable knockdown of FoxM1 were generated by transducing Hep3B cell line with either pSuper or shFoxM1-puromycin based retroviral vector (shFoxM1 sense: 5’- GATCCCCGGAAATGCTTGTGATTCAATTCAAGAGATTGAATCACAAGCATTTCCTTTTTA- 3’ and anti-sense: 5’- AGCTTAAAAAGGAAATGCTTGTGATTCAATCTCTTGAATTGAATCACAA GCATTTCCGGG- 3’). Pure, virally transduced population was selected and maintained in media containing puromycin (3 µg/ml). FoxM1 knockdown was verified by assessing the expression of endogenous FoxM1 using western blotting.

### RT-PCR

Total RNA was isolated using Trizol reagent (Invitrogen, Carlsbad, CA), according to the manufacturer’s instructions. One microgram of total RNA was used to synthesize cDNA with SuperScript II Reverse Transcriptase (Invitrogen, Carlsbad, CA). Real-time PCR was performed utilizing the SYBR GREEN Master Mix (Applied Biosystems, Foster City, CA, USA) as per the manufacturer’s protocol with GAPDH as internal control. Following sets of primers were used:

FoxM1 (sense): 5’- GGAGGAAATGCCACACTTAGCG- 3’ and (anti-sense): 5’- TAGGACTTCTTGGGTCTTGGGGTG- 3’,
Plk1 (sense): 5’- ATCACCTGCCTGACCATTCCAC- 3’ and (anti-sense): 5’- TCTCCAAGCCTTTATTGAGGACTG- 3’,
CyclinB1 (sense): 5’- CGGGAAGTCACTGGAAACAT- 3’ and (anti-sense): 5’- AAACATGGCAGTGACACCAA- 3’,
Skp2 (sense): 5’- GGTGTTTGTAAGAGGTGGTATCGC- 3’ and (anti-sense): 5’- CACGAAAAGGGCTGAAATGTTC- 3’,
Aurora B kinase (sense): 5’- TCACACAACGAGACCTATCGCC- 3’ and (anti-sense): 5’- GGGGTTATGCCTGAGCAGTTTG- 3’,
GAPDH (sense): 5’- ACCTGACCTGCCGTCTAGAA- 3’ and (anti-sense): 5’- TCCAACCACCCTGTTGCTGTA- 3’.

### Cycloheximide Chase Assay

Protein degradation assay was performed as elaborated previously (48). In brief, cells were treated with 100 µg/ml cycloheximide (Sigma, St. Louis, MO), harvested at varied time intervals and equal amounts of the whole cell lysates were subjected to western blot analysis. Densitometric analyses of scanned images were carried out using ImageJ software.

### Nuclear-Cytoplasmic Fractionation

Cells were harvested in PBS containing 4 mM EDTA and suspended in hypotonic buffer (10 mM HEPES pH 7.9, 1.5 mM MgCl_2_, 10 mM KCl, 2 mM PMSF, 2 mM NaF, 1 mM Na_3_VO_4_ and protease inhibitor cocktail) and incubated on ice for 45 mins followed by dounce homogenization. Cells were centrifuged at 2000 rpm for 10 mins at 4°C and supernatant was collected as the cytoplasmic fraction. The pellet was washed with hypotonic buffer, rocked at 4°C for 10 mins and centrifuged. Thereafter, it was suspended in hypertonic buffer (20 mM HEPES pH 7.9, 1.5 mM MgCl_2_, 500 mM KCl, 0.2 mM EDTA, 20% glycerol and protease inhibitor cocktail), rocked at 4°C for 45 mins, centrifuged at high speed and the supernatant was retained as the nuclear fraction. Purity of the fractions was verified using specific cytoplasmic (GAPDH) and nuclear (PARP) markers.

### Chromatin Immunoprecipitation (ChIP)

Cells were washed with PBS, cross-linked with 1% formaldehyde at RT for 20 mins and the reaction was quenched with 20 mM glycine. Following wash with PBS, cells were incubated in lysis buffer (50 mM Tris-HCl pH 8.1, 10 mM EDTA pH 8.0, 1% SDS and protease inhibitor cocktail) and sonicated to shear the chromatin into small fragments. The sheared chromatin DNA mixture (normalized input) was incubated with 1 μg of anti-FoxM1 overnight at 4°C. Anti-rabbit IgG (1 μg) was used as a negative control. Following morning, the immune complexes were captured through pre-blocked protein A bead slurry for 3 h at 4°C, followed by multiple washes (1.665 mM Tris- HCl pH 8.1, 1.2 mM EDTA pH 8.0, 150 mM NaCl, 0.01% SDS and 1% Triton) and elution (50 mM NaCl, 100 mM NaHCO_3_ and 1% SDS). The eluted complex along with the input was reverse cross- linked overnight at 65°C. Subsequently, samples were precipitated using chilled ethanol and sodium acetate pH 5.2 (frozen at -80°C for 3 h) and washed once with 70% chilled ethanol. Once dry, the DNA pellet was treated with proteinase K for 45 mins at 37°C followed by phenol chloroform extraction. Samples were precipitated with chilled ethanol and sodium acetate pH 5.2, washed in 70% chilled ethanol. After subsequent drying, the pellet was suspended in freshly autoclaved water. PCR was carried out using the following primer sets:

Plk1 (sense): 5’- AGGAGGGGAAGGTGAGGAAA- 3’ and (anti-sense): 5’- GAGAAGCATTTGGGGGAGGG- 3’,
Aurora B Kinase (sense): 5’- GCAACGAAAGGTCTATTGGTGG- 3’ and (anti-sense): 5’- TCTAACTTCTCTGCCCGATGGAG- 3’,
Cdc25B (sense): 5’- AAGAGCCCATCAGTTCCGCTTG- 3’ and (anti-sense): 5’- CCCATTTTACAGACCTGGACGC- 3’
CyclinB1 (sense): 5’- CGCGATCGCCCTGGAAACGCA- 3’ and (anti-sense): 5’- CCCAGCAGAAACCAACAGCCGT- 3’
GAPDH (sense): 5’- AAAAGCGGGGAGAAAGTAGG- 3’ and (anti-sense): 5’- CTAGCCTCCCGGGTTTCTCT- 3’.

### Electrophoretic Mobility Shift Assay (EMSA)

EMSA was performed using a Cy5-labeled oligo (5’-Cy5-AAACAAACAAACAAACAAACAAACAATC- 3’), comprising of the consensus promoter sequence recognized by FoxM1, was commercially synthesized (IDT, Coralville, IA, USA). The purified DBD of FoxM1b (1.5 µg) [method described in (49)] was mixed with the Cy5- labeled DNA probe (25 pmole) in a 20 µl reaction mixture, containing a final concentration of 20 mM Tris-HCl pH 7.5, 100 mM KCl, 1 mM MgCl_2_, 10% glycerol, 0.01 mg/ml bovine serum albumin and 0.1 M DTT (Sigma, St. Louis, MO) and incubated at RT for 1 h. The specificity of the FoxM1-DBD/DNA complex was determined with a 100X unlabeled oligo. For displacement experiments, increasing concentrations of Artemisinin were added to the reaction mixture. The reactions were resolved in 4% native polyacrylamide gel electrophoresis in 0.5X TBE buffer at 125 volts at 4°C. Images were captured on a FLA-9000 image analyzer.

### Immunostaining and Fluorescence Microscopy

Cells were seeded and cultured on coverslips followed by fixing in 4% *para*-formaldehyde solution (w/v) in PBS for 20 mins at RT prior to washing with PBS. Permeabilization was carried out in PBS containing 0.5% triton for 10 mins at RT followed by blocking with 5% bovine serum albumin (w/v) for 1 h. Overnight incubation with anti-FoxM1 (1:500) was followed by washing with PBS and incubation with Alexa Fluor 488-conjugated anti-rabbit secondary antibody (Cat #A-11094, RRID : AB_221544, 1:1000) (Invitrogen, Carlsbad, CA) for 1 h at RT. Following washes with PBS, nuclei were counterstained with 4’, 6-diamidino-2-phenylindole (DAPI; Invitrogen, Carlsbad, CA). Coverslips were mounted on microscopic slides and images were acquired using fluorescence microscope (Nikon-Eclipse-Ti-S, Tokyo, Japan).

### BrdU Immunofluorescence

BrdU (bromodeoxyuridine) assay was carried out to measure the amount of proliferating cells wherein cells were incubated with BrdU (Sigma, St. Louis, MO) solution for 1 h at 37°C. Cells were washed with PBS and fixed in 4% *para*-formaldehyde. Permeabilization in PBS containing 0.1% triton was followed by incubation with 1 (N) HCl on ice for 10 mins and 2 (N) HCl at RT for 10 mins. Phosphate citric acid buffer pH 7.4 was added and cells were incubated at RT for 10 mins prior to washes with PBS containing 0.1% triton. Cells were blocked in 5% bovine serum albumin for 1 h at 4°C and incubated overnight with anti-BrdU (1:500) (Cat #RPN202, RRID : AB_2314032, GE Healthcare, Buckinghamshire, UK) at 4°C. Following day, cells were incubated with Alexa Fluor 594-conjugated anti-mouse secondary antibody (Cat #A-11032, RRID : AB_2534091, 1:1000) (Invitrogen, Carlsbad, CA) for 1 h at RT. Cells were washed with PBS and nuclei were counterstained with DAPI. Cells were mounted on slides and observed under fluorescence microscope (Nikon-Eclipse-Ti-S, Tokyo, Japan). The number of BrdU positive cells was counted and graphically presented relative to the control.

### TUNEL Assay

The terminal deoxynucleotidyltransferase-mediated dUTP nick end labeling (TUNEL) method was executed to label the 3ʹ-end of fragmented DNA of apoptotic cells. Cells seeded with coverslips were fixed in 4% *para*-formaldehyde followed by permeabilization in PBS containing 0.1% triton. Cells were washed with deionized water thrice and subjected to Click-iT™ Plus TUNEL assay (Invitrogen, Carlsbad, CA) as per manufacturer’s instructions. Nuclei were counterstained with DAPI and the coverslips were mounted onto slides. Images were captured using a fluorescence microscope (Nikon-Eclipse-Ti-S, Tokyo, Japan) and the percentage of TUNEL positive cells was counted and graphically represented with respect to the control.

### JC-1 Assay

The MitoProbe™ JC-1 assay kit (Molecular Probes, Eugene, OR) was employed for detection of mitochondrial membrane potential, which collapses during cellular apoptosis. The electrochemical potential gradient of 5, 5’, 6, 6’ tetrachloro-1, 1’, 3, 3’-tetraethyl benzimidazolocarbocyanine iodide (JC-1) allows it to accumulate in the mitochondrial matrix in normal cells, where the dye exists as red fluorescent J-aggregates. Its mitochondrial localization is disrupted upon dissipation of the mitochondrial membrane potential wherein JC-1 disperses throughout the entire cell due to formation of green fluorescent J-monomers, implying depolarization of the mitochondrial membrane. A higher red to green fluorescence ratio indicates cell viability whereas a lower ratio suggests apoptosis. Briefly, cells were washed with PBS and incubated in medium containing JC-1 (final concentration of 2 µM) for 30 mins in a 37°C humidified CO_2_ incubator. This media was replaced with PBS and cells were immediately photographed using a fluorescence microscope (Nikon-Eclipse-Ti-S, Tokyo, Japan). The fluorescence intensities of the aggregate and monomeric forms were measured at excitation/emission wavelengths (550 nm/600 nm for red) and (485 nm/535 nm for green), respectively, using Varian Cary Eclipse spectrophotometer and the ratio was expressed relative to control set.

### Cell Viability Assay

The rate of metabolically active cells was evaluated using MTT assay, according to the manufacturer’s protocol. Cells were seeded at a density of 8x10^3^ cells/well in 96-well plates (Corning Inc., Corning, NY) and cultured with different treatments for defined duration at 37°C in a humidified CO_2_ incubator. Cells were incubated with solution of 3-(4,5-dimethylthiazol-2-yl)-2,5-diphenyltetrazolium bromide (MTT; Sigma, St. Louis, MO) for 4 h. Supernatant was removed and dimethyl sulphoxide (Sigma, St. Louis, MO) was added to dissolve the purple formazan crystals. The absorbance was measured at 570 nm with a microplate reader (Tecan microplate reader, Infinite M200 Pro). Cell viability was estimated as a percentage of the value of the untreated control cells.

### Cell Proliferation Analysis

Cells were seeded at the density of 2000 cells/well in 24-well plates (Corning Inc., Corning, NY) followed by appropriate treatments as mentioned. Every alternate day, cells were washed, trypsinized and counted. Rate of cell proliferation for the given days was calculated relative to vehicle treated set.

### Colony Formation and Soft Agar Assay

Clonogenic potential of cells was measured using colony forming assay wherein cells were seeded at 1500 cells/well in 24-well plates (Corning Inc., Corning, NY). Cells were given respective treatments and allowed to grow in a humidified 37°C CO_2_ incubator. Media was changed every 3-4 days for 14 days when colonies of appreciable size (≥50 cells) were obtained. Colonies were, thereafter, fixed in methanol and stained with 0.5% crystal violet stain (Sigma, St. Louis, MO), photographed, counted and the number of colonies was graphically presented compared to control.

Anchorage-independent colony forming ability of cells was assessed through soft agar assay wherein 1x10^4^ cells were suspended in medium, containing appropriate concentrations of drug and 0.8% agar and then poured onto the wells of 6-well plates (Corning Inc., Corning, NY) coated with medium containing 1.6% agar. The top agar surface was layered with complete medium every third day and cells were allowed to grow for 20-30 days. When colonies became larger than 0.1 mm in soft agar, they were stained with 0.1% crystal violet for capturing image and subsequent counting. The size of the colonies was graphically shown relative to the control.

### Migration and Invasion Assays

Cells were seeded to complete confluence in a monolayer in 24-well plates (Corning Inc., Corning, NY). A wound was created by scratching firmly with a 20 µl tip and baseline (time zero) images were captured. Cells were incubated at 37°C in a humidified CO_2_ incubator and photographed at the indicated time-points. The width of the injury line remaining was measured against each time-point and graphically depicted as percent wound relative to the control set.

Invasion assay was performed using 24-well BD BioCoat Matrigel invasion chambers (BD Biosciences, San Jose, CA), according to the manufacturer’s instructions. Briefly, inserts were rehydrated using serum free media (Invitrogen, Carlsbad, CA) for at least 2 h. A total of 1x10^5^ cells were seeded in the upper inserts in 0.5 ml of serum free medium while the bottom wells were filled with complete media. Cells were allowed to invade towards the complete medium, which acts as a chemoattractant, through the matrigel-coated membrane for 48 h in a 37°C CO_2_ incubator. Non-invading cells were removed using a damp cotton swab while the invading cells that adhered to the bottom surface of the insert were fixed in methanol, stained with 0.5% crystal violet stain and counted under a light microscope and graphically presented compared to the control.

### Determination of Drug-Drug Interaction

The fractional inhibitory concentration (FIC) was calculated for each drug ratio (4:1, 3:2, 2:3 and 1:4), using the IC_50_ values, and the FIC_50_ was determined with the following equation:


$$⅀\;FIC_{50}=\;\left(IC_{50}of\;Artemisinin\;in\;combination/IC_{50}\;of\;Artemisinin\;alone\right)\;+\;\left(IC_{50}of\;Thiostrepton\;in\;combination/IC_{50}of\;Thiostrepton\;alone\right)$$

An overall mean ⅀ FIC_50_ value for each combination was evaluated and synergy or antagonism was defined as a mean ⅀ FIC_50_ < or > 1, respectively, while additivity was defined as ⅀ FIC_50_ = 1, as done previously (50).

### Statistical Analysis

All data are representative of at least three independent experiments. Results were expressed as means ± standard errors of the means (SEM). The two-tailed Student’s *t*-test was used to determine a significant difference between two independent groups. The difference between groups was set at *p < 0.05, **p < 0.01 and ***p < 0.001.

## Results

### Artemisinin Sharply Reduces HCC Cell Viability and Proliferation

**Figure 1:**
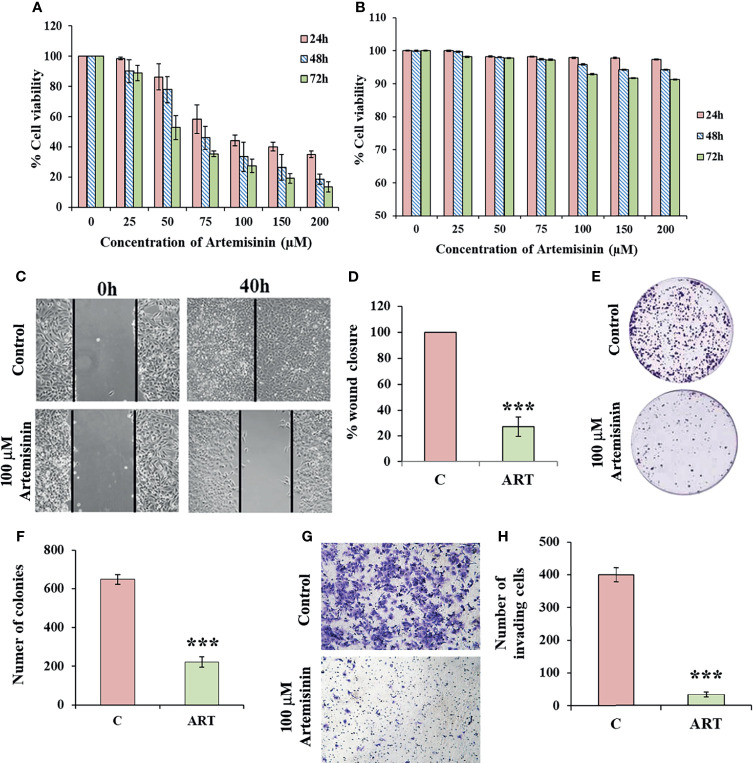
Artemisinin mitigates HepG2 tumorigenic potential. **(A)** The percent cell viability from MTT assay of HepG2 cells treated with increasing concentrations of Artemisinin (25, 50, 75, 100, 150 and 200 µM) for 24, 48 and 72 h or vehicle is graphically presented. **(B)** Percent cell viability of HEK293T cells exposed to varying concentrations of Artemisinin or vehicle followed by assessment using MTT assay is shown. **(C)** Scratch assay was performed in cells exposed to 100 µM Artemisinin for 40 h. Representative images of the scratched area at the indicated time-points are presented **(D)** Percent wound closure is graphically shown. **(E)** Colony formation assay was executed in cells exposed to 100 µM Artemisinin for 48 h. Representative images of stained colonies are shown. **(F)** The measured number of colonies is graphically represented. **(G)** Matrigel invasion assay was performed in cells following exposure to 100 µM Artemisinin for 48 h. Representative images of stained cells are provided. **(H)** Graph showing the number of invasive cells. ***p < 0.001. All data are expressed as the means ± standard deviations of three independent experiments. The two-tailed Student’s *t*-test was used to determine whether the differences between vehicle-treated set and Artemisinin-treated set were significant.

For assessing the cytotoxicity of Artemisinin towards HCC cells, we performed cell viability assay in HepG2 cells. MTT assay revealed profound cell death following Artemisinin treatment in dose- and time-dependent manners (**Figure 1A**). On contrary, no inhibitory effect was seen on the growth of human embryonic kidney cell line, HEK293T, at the same concentrations of Artemisinin (**Figure 1B**). This palpably indicated the selective action of Artemisinin on hepatic cancer cells without posing any drastic toxicity to non-cancerous cells. The ability of cancer cells to hyper-proliferate is a measure of tumorigenicity *in vitro*, which prompted us to test for the influence of Artemisinin on HepG2 cell proliferation. Results from scratch assay demonstrated that Artemisinin significantly (four-fold) inhibited HepG2 cell migration (**Figures 1C, D** **)**, as evident from the intact wound visible in Artemisinin-treated cells even at 40 h. A major concern for most HCC patients is tumor relapse after completion of anticancer therapy. We, therefore, performed colony formation assay to investigate the outcome of Artemisinin on long-term clonogenic ability of HepG2 cells. Interestingly, incubation with Artemisinin for 48 h was sufficient to decrease the frequency and size of HepG2 cell colonies by three-fold (**Figures 1E, F** **)**, alluding that Artemisinin-treated cancer cells fail to regain their ability to grow and form colonies of substantial size even after drug removal. Moreover, the invasive index of tumor cells renders them the exceptional quality to metastasize. In agreement with our previous observations, Artemisinin yielded a remarkable decline in the invasive capability of HepG2 cells by eight-fold (**Figures 1G, H** **)**. Altogether, Artemisinin effectively suppressed various neoplastic properties of HepG2 cells with negligible cytotoxicity to healthy cells, implying its utility as a promising anticancer therapy.

### Artemisinin Attenuates FoxM1 and Its Transcriptional Targets

**Figure 2:**
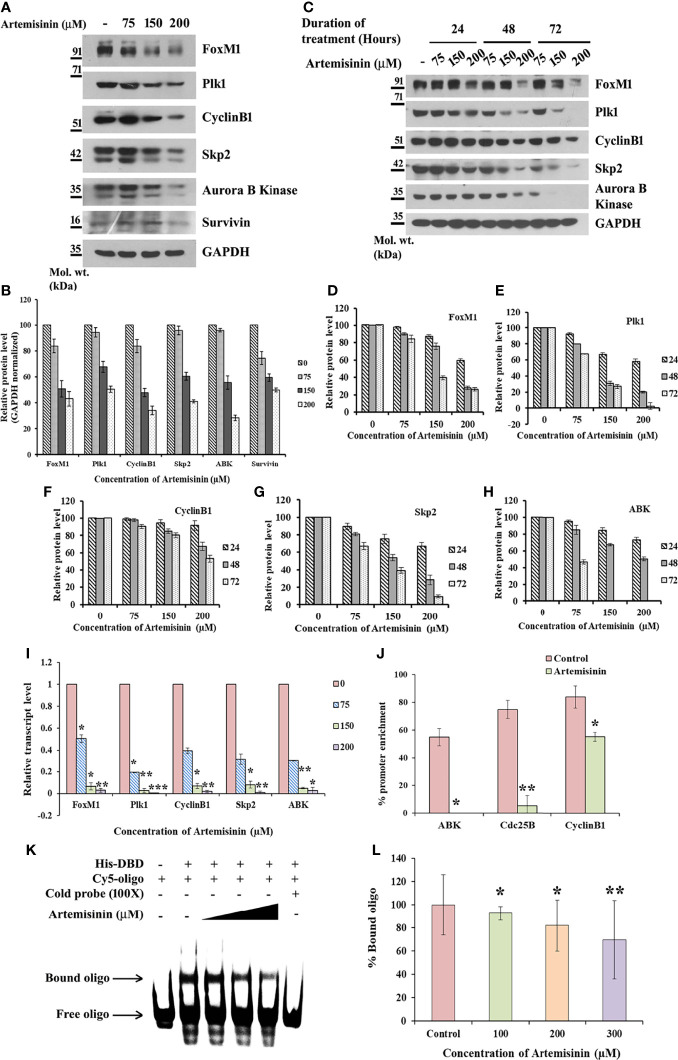
Artemisinin impedes FoxM1 transcriptional activity. **(A)** Equal amounts of HepG2 cell lysates treated with Artemisinin for 48 h were subjected to SDS-PAGE and immunoblotting using antibodies specific for FoxM1, Plk1, CyclinB1, Skp2, Aurora B Kinase and Survivin. GAPDH was used as a loading control. **(B)** Densitometric analysis of the relative protein levels of FoxM1 antibody and its targets using ImageJ, normalized to GAPDH. **(C)** HepG2 cells were treated with Artemisinin for 24, 48 and 72 h followed by SDS-PAGE and western blotting for FoxM1 and its downstream effectors with GAPDH as a loading control. **(D–H)** Graphical representation of the relative protein levels of FoxM1 and its targets quantified using ImageJ. **(I)** Total RNA was isolated from HepG2 cells treated with Artemisinin using TriZOL. qRT-PCR was performed using SYBR Green assay. The fold mRNA change has been represented graphically, normalized to GAPDH. **(J)** HepG2 cells were treated with 100 µM Artemisinin for 24 h followed by ChIP using anti-FoxM1 anti-body and PCR with gene-specific primers. GAPDH served as an internal control. The percent promoter occupancy by FoxM1 is represented graphically. **(K)** Purified FoxM1-DBD was mixed with the Cy5-labeled DNA probe and Artemisinin added to the reaction in increasing concentrations. Representative EMSA image is shown. **(L)** Quantification of the percent bound oligo is presented. *p < 0.05, **p < 0.01, and ***p < 0.001. All data are expressed as the means ± standard deviations from triplicate experiments. The two-tailed Student’s *t*-test was used to determine whether the differences between vehicle-treated set and Artemisinin-treated set were significant.

Considering the pivotal involvement of FoxM1 up-regulation during HCC tumor development and progression (32, 51, 52), we sought to gain insight into the possible regulatory effect of Artemisinin on FoxM1. Artemisinin treatment in HepG2 cells led to a marked dosewise reduction in the levels of FoxM1 and its major transcriptional targets, such as Plk1, CyclinB1, Skp2, Aurora B Kinase and Survivin (**Figures 2A–H**). We next addressed the important relevance of augmented levels of FoxM1 and its principle targets with overall survival in HCC patients. Of note, high expression of FoxM1 and its downstream effector factors (Plk1, CyclinB1, Skp2 and Aurora B Kinase) correlated with poor overall survival in hepatic cancer patients (**Supplementary Figures 1A–E**, analyzed from RNA-seq data available on Kaplan-Meier plotter) (53). Hence, our findings imply that Artemisinin may exert its tumor suppressive effect in HCC cells by down-regulating FoxM1 and its downstream target genes.

### Artemisinin Induces Transcriptional Inhibition of FoxM1

Next, cycloheximide half-life assay revealed no remarkable difference in the protein turnover of FoxM1, Plk1, CyclinB1, Skp2 and Aurora B Kinase following Artemisinin treatment (**Supplementary Figure 2A**). This suggests that Artemisinin does not alter *de novo* protein synthesis of these factors. Evidence from existing literature indicates that FoxM1 nuclear translocation in hepatocytes stimulates cells to proliferate (54) and being a transcriptional factor, its nuclear localization is a pre-requisite for its downstream functions. To address whether Artemisinin modulates the sub-cellular distribution of FoxM1, HepG2 cells treated with either control or 100 µM Artemisinin for 48 h were subjected to nuclear cytoplasmic fractionation. Western blot analysis of the fractions did not exhibit any noticeable change in FoxM1 cellular translocation although there was an overall drop in its level in response to Artemisinin (**Supplementary Figure 2B**). This was further corroborated through immunostaining for FoxM1 wherein although a similar reduction in FoxM1 expression was achieved in presence of Artemisinin (**Supplementary Figure 2C**, lower panel), there was no variation in its localization. These results drove us to consider the possibility of Artemisinin’s effect on FoxM1 transcriptional level. As shown in **Figure 2I**, qRT-PCR of HepG2 cells treated with increasing doses of Artemisinin displayed a sharp decline in the transcript levels of FoxM1 and its principle targets, Plk1, CyclinB1, Skp2 and Aurora B Kinase, in a concentration-related manner. Our data, thus, denoted that Artemisinin stifled expression of FoxM1 and its transcriptional targets at their transcript levels.

### Artemisinin Interrupts FoxM1 *Trans*-activating Ability

FoxM1 inhibitors like Thiostrepton and FDI-6 are reported to abolish the *trans*-activation of its targets (55, 56). To determine whether Artemisinin perturbs FoxM1’s ability to bind to its native consensus promoter regions, ChIP was executed in HepG2 cells. While amelioration in the FoxM1 promoter occupancy of its downstream target genes, Aurora B Kinase, Cdc25B and CyclinB1, was prominent in the vehicle treated set, FoxM1 inhibition by Artemisinin abrogated this enrichment by an appreciable fold (**Figure 2J** and **Supplementary Figure 3**). The results confirmed that Artemisinin directly suppresses the transcriptional activity of FoxM1. Furthermore, EMSA of the complex formed between purified FoxM1-DBD and a Cy5-labeled oligo comprising of the putative DNA-binding motif of FoxM1 in the presence of Artemisinin led to a considerable (almost 30%, *p* < 0.01) decrease in the bound form of FoxM1-DBD (**Figures 2K, L** **)**. These data strongly indicate that Artemisinin disrupts FoxM1’s *trans*-activation function to repress the expression of its oncogenic target genes, thus depicting a novel tumor suppressive mechanism of Artemisinin.

### FoxM1 Knockdown Sensitizes Resistant HCC Cells to Artemisinin

**Figure 3:**
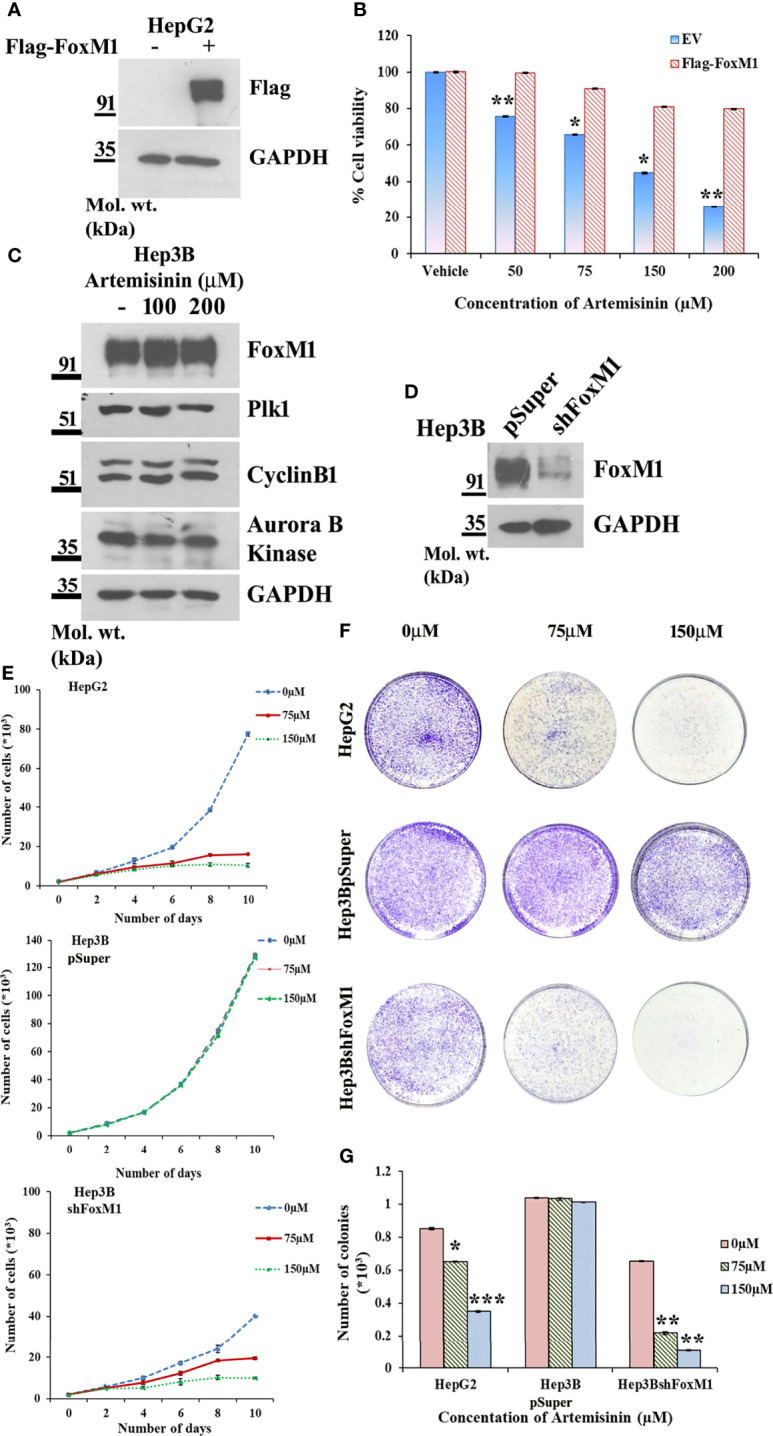
FoxM1 level contributes to Artemisinin-mediated mitigation of cancerous properties of HCC cells. **(A)** HepG2 cells were transiently transfected with either empty vector or Flag-FoxM1. Equal amount of whole cell lysate was subjected to SDS-PAGE and subsequent immunoblotting for FoxM1. GAPDH was used as a loading control. **(B)** HepG2 cells overexpressing either empty vector or Flag-FoxM1 were treated with increasing doses of Artemisinin for 48 h and cell viability was examined using MTT assay. **(C)** Hep3B cells were treated with either vehicle or Artemisinin (100 and 200 µM) for 48 h. Equal amounts of whole cell lysates were subjected to SDS-PAGE and western blotting with antibodies specific for FoxM1 and its transcriptional targets, such as Plk1, cyclinB1 and Aurora B kinase. GAPDH was used as a loading control. **(D)** Immunoblotting showing the efficiency of FoxM1 knockdown in Hep3BshFoxM1 stable cell line relative to control Hep3BpSuper cells. **(E)** The comparative proliferative rates of HepG2, Hep3BpSuper and Hep3BshFoxM1 cells following exposure to Artemisinin for 48 h. **(F)** Representative images and **(G)** graphical presentation of the stained colonies formed by Artemisinin-treated cells after clonogenic assay. *p < 0.05, **p < 0.01 and ***p < 0.001. All data are expressed as the means ± standard deviations from triplicate experiments. The two-tailed Student’s *t*-test was used to determine whether the differences between vehicle-treated set and Artemisinin-treated set were significant.

Augmented FoxM1 level reportedly causes a higher likelihood of resistance to anticancer therapy whereas targeting FoxM1 reversed this phenotype (57–60). To further strengthen this notion, Flag-FoxM1 expression plasmid was ectopically expressed in HepG2 cells (**Figure 3A**). MTT assay revealed that when these FoxM1-overexpressing cells were treated with increasing doses of Artemisinin for 48 h, there was negligible cytotoxicity observed in these cells (**Figure 3B**). This was in stark contrast to what we noticed when parental HepG2 cells were treated with Artemisinin (**Figure 1A**). This indicated that FoxM1 overexpression in sensitive HCC cells may promote resistance in them towards anticancer therapy. Our next goal was to determine whether silencing of FoxM1 renders resistant HCC cells susceptible towards Artemisinin therapy. The Hep3B hepatoma cells, which are known to be sorafenib-resistant (61, 62), was previously shown to exhibit elevated level of FoxM1 (41). Importantly, we observed no substantial suppression in the level of FoxM1 and its downstream targets, such as Plk1, cyclinB1 and Aurora B kinase upon treatment of Hep3B cells with increasing concentrations of Artemisinin (**Figure 3C**). This was in sharp contrast to our results in HepG2 cells (**Figures 2A–H**). In accordance, to investigate whether resistance of Hep3B cells to contemporary therapeutic regimes was due to elevated FoxM1 level, we stably silenced FoxM1 in Hep3B cells (**Figure 3D**). Our western blotting further substantiated earlier observations as a distinctly higher FoxM1 level was noted in Hep3BpSuper cells as opposed to HepG2 cells (**Supplementary Figure 4**). Following treatment with Artemisinin, Hep3BshFoxM1 cells revealed a considerable diminution in their proliferative index while Hep3BpSuper cells remained unaffected (**Figure 3E**). In sync, the growth rate of Hep3BshFoxM1 cells mimicked the pattern demonstrated by the sensitive HepG2 cells. Similarly, a substantial decrease (three-fold) in the colony forming proficiency of FoxM1 silenced Hep3B cells was observed with no apparent effect on parental Hep3B cells (**Figures 3F, G** **)**.

**Figure 4:**
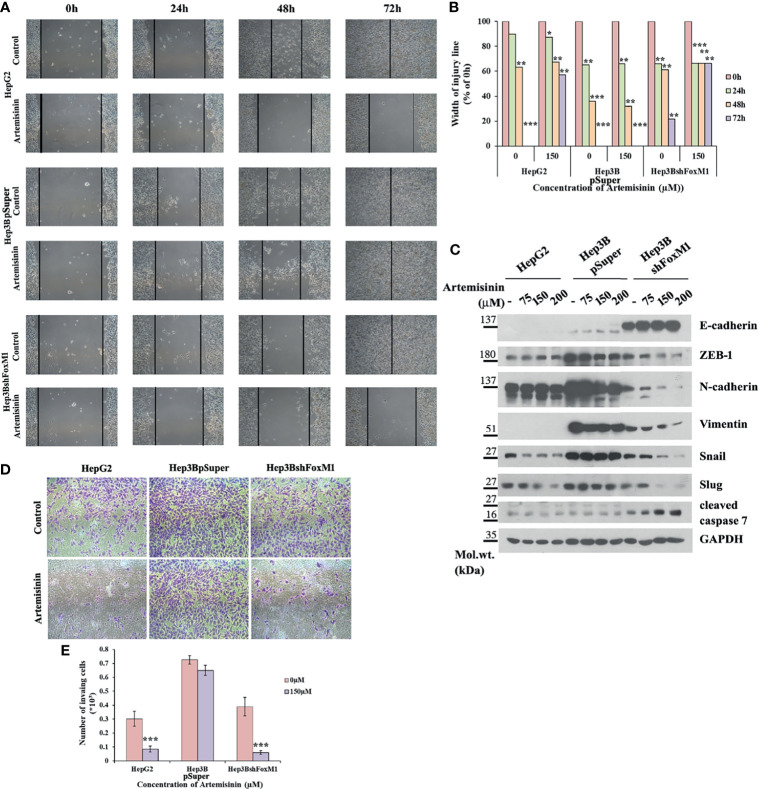
Artemisinin attenuates the transformative capacity of resistant HCC cells following FoxM1 silencing. **(A)** HepG2, Hep3BpSuper and Hep3BshFoxM1 cells were subjected to scratch assay. Representative images of gap closure are shown. **(B)** The width of the wound remaining was measured and graphically depicted. **(C)** Equal amounts of cell lysates following Artemisinin treatment for 48 h were subjected to SDS-PAGE and immunoblotted with antibodies specific for E-cadherin, ZEB-1, N-cadherin, Vimentin, Snail, Slug and cleaved caspase 7. GAPDH served as a loading control. **(D)** Matrigel invasion assay was executed for cells following 48 h exposure to Artemisinin. Representative images are provided. **(E)** Bar graph depicting the number of invading cells. *p < 0.05, **p < 0.01, and ***p < 0.001. All data are expressed as the means ± standard deviations from triplicate experiments. The two-tailed Student’s *t*-test was used to determine whether the differences between vehicle-treated set and Artemisinin-treated set were significant.

The conjecture was further reinforced by analysis of cell migration using wound healing assay, wherein Hep3BshFoxM1 cells displayed a marked delay in wound closure with more than 65% intact gap remaining after 72 h treatment with Artemisinin compared to parental Hep3B cells that showed complete wound closure (**Figures 4A, B** **)**. Previous research has shown FoxM1 to regulate the expression of epithelial to mesenchymal transition (EMT)-related genes, thereby accelerating carcinogenesis (63). Considering EMT as a pivotal feature of cancer cells that render them with the dexterity to metastasize, we sought to explore the role of FoxM1 silencing in curbing this phenomenon in the resistant HCC cells. Our immunoblotting denotes a dose-dependent rise in level of the epithelial marker, E-cadherin, in Hep3BshFoxM1 but not in Hep3BpSuper cells, as depicted in **Figure 4C**. In contrast, mesenchymal markers, N-cadherin, Vimentin, ZEB-1, Snail and Slug, displayed a clear drop in Artemisinin-treated Hep3BshFoxM1 cells (**Figure 4C**), insinuating that the metastatic potential of Hep3B cells was reversed in response to Artemisinin upon FoxM1 knockdown. Furthermore, there was an increased induction of cleaved caspase 7 in Artemisinin-exposed Hep3BshFoxM1 cells, implying an enhanced apoptosis in these cells. For further evaluation, we performed Transwell invasion assay, which revealed a significant four-fold (*p* < 0.001) alleviation in the invasive nature of FoxM1 silenced Hep3B cells in response to Artemisinin treatment (**Figures 4D, E** **)**, similar to HepG2 cells. No severe change was noticed in the invasiveness of control Hep3BpSuper cells.

To procure further insight into the mechanism of FoxM1-mediated resistance in Hep3B cells, we performed TUNEL assay and measured the extent of apoptosis. Loss of apoptotic regulation provides cancer cells the opportunity for longer survival and more time for additional accumulation of mutations, thereby facilitating tumor advancements (64). Both the FoxM1 depleted Hep3B cells and sensitive HepG2 cells exhibited noteworthy increase (80%, *p* < 0.001) in cell death following treatment with Artemisinin compared to resistant line (**Supplementary Figure 5**), implying the regained ability of Hep3BshFoxM1 cells to undergo apoptosis following anticancer treatment. Cumulatively, our results indicated that FoxM1 silencing in resistant HCC cells effectively sensitized them to the tumor suppressive activities of Artemisinin, reflected by their attenuated neoplastic transformations and enhanced apoptosis.

### Thiostrepton Potentiates the Tumor Suppressive Activity of Artemisinin

**Figure 5:**
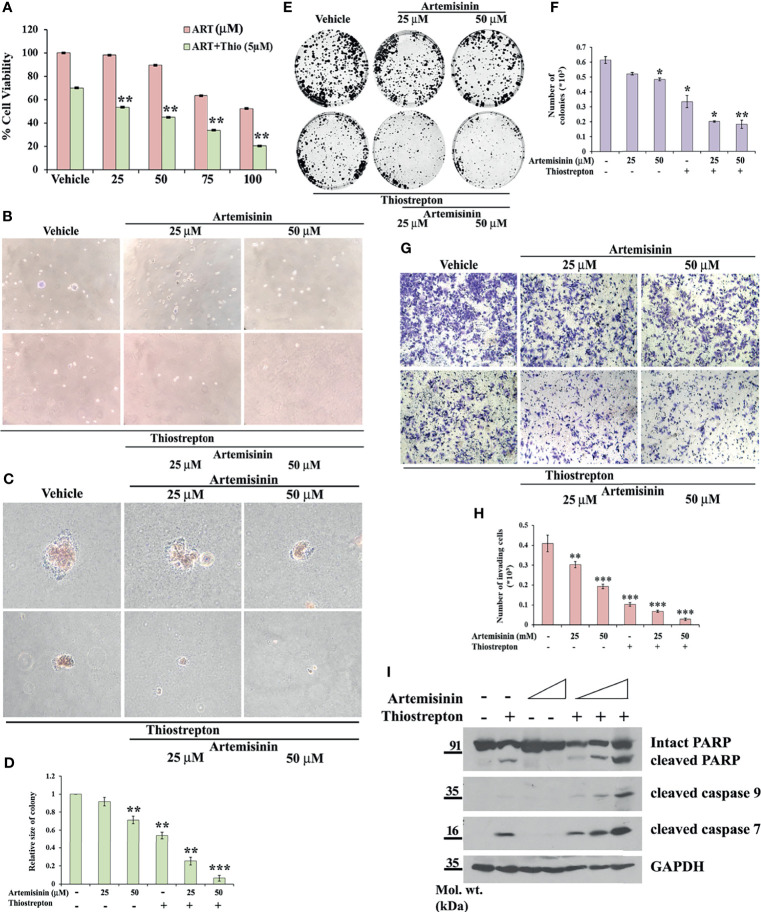
Thiostrepton enhances Artemisinin-mediated growth inhibition. **(A)** Cell viability was measured in HepG2 cells treated with indicated concentrations of Artemisinin and Thiostrepton for 48 h. **(B)** Representative images of colonies formed in soft agar by HepG2 cells treated with Artemisinin alone or in conjunction with Thiostrepton for 48 h at 20X and **(C)** 40X magnification using an inverted microscope are shown. **(D)** Graphical depiction of the relative size of colonies. **(E)** Representative images of colony formation assay of HepG2 cells treated with Artemisinin alone or in combination with Thiostrepton for 48 h are presented. **(F)** Graphical representation of the number of colonies. **(G)** Representative images of invading cells following matrigel invasion assay performed in HepG2 cells treated with Artemisinin alone or in combination with Thiostrepton are provided. **(H)** Bar graph depicting the number of invading cells. **(I)** HepG2 cells treated with Artemisinin alone (25 and 50 µM) or in combination (12.5, 25, 50 µM) with Thiostrepton for 48 h were immunoblotted for cleaved PARP, cleaved caspase 9 and cleaved caspase 7. GAPDH served as a loading control. *p < 0.05, **p < 0.01, and ***p < 0.001. All data are expressed as the means ± standard deviations from triplicate experiments. The two-tailed Student’s *t*-test was used to determine whether the differences between vehicle-treated set and Artemisinin-treated set were significant.

Although our findings illustrated the significant efficacy of Artemisinin as a single agent, we wished to explore its therapeutic response in conjunction with established chemotherapeutics. Since our findings denoted FoxM1 as a pivotal target of Artemisinin, we thought of exploiting an established FoxM1 inhibitor, Thiostrepton (55) (**Supplementary Figure 6A**), to interrogate the combined anticancer efficacy of both drugs at sub-optimal doses. To evaluate the anti-tumor effects of Artemisinin and Thiostrepton, we first investigated their combined outcome on HepG2 cell viability. As shown in **Figure 5A**, co-treatment of Artemisinin with Thiostrepton evidently enhanced the growth inhibitory effect of Artemisinin on HepG2 cells. Of note, our cell viability assay using various ratios of combinations of Artemisinin and Thiostrepton revealed a mean ⅀ FIC_50_ of 0.9823 ± 0.1802 (mean value ± standard deviation). Thus, our data suggests a slightly synergistic to a nearly additive interaction with Artemisinin and Thiostrepton. Intriguingly, western blot analysis of HepG2 cells clearly showed that while Artemisinin, on its own, at lower doses (25 and 50 µM), did not effectively downregulated FoxM1 level, a prominent decline in FoxM1 level was visible in the presence of lower therapeutic dosage of Artemisinin, upon its combination with Thiostrepton (**Supplementary Figure 6B**).

Our next goal was to gauge the anti-carcinogenic potential of Artemisinin at lower doses in juxtaposition with Thiostrepton. We investigated the outcome of the combined treatment on anchorage-independent colony forming capacity of HepG2 cells. Treatment with 25 µM of Artemisinin alone did not yield sufficient change in the colonies formed in soft agar while incubation with 50 µM of Artemisinin alone resulted in moderate (1.4-fold) decrease in the size and number of colonies formed on soft agar by HepG2 cells. Although Thiostrepton alone impeded both the number and size of the colonies by two-fold, the effect was accentuated (more than five-fold) in presence of combination therapy (**Figures 5B–D**). Next, colony formation assay divulged that the robust colony forming ability of HepG2 cells was moderately repressed by the low doses of Artemisinin and extensively inhibited by Thiostrepton (two-fold), when used alone. However, this was diminished even further (three to four-fold) upon co-treatment (**Figures 5E, F** **)**. The combination was further assessed for its influence on cellular invasion. In agreement with our previous observations, co-administration of Thiostrepton and Artemisinin drastically hindered the invasive character of HepG2 cells by more than five-fold (**Figures 5G, H** **)**.

**Figure 6:**
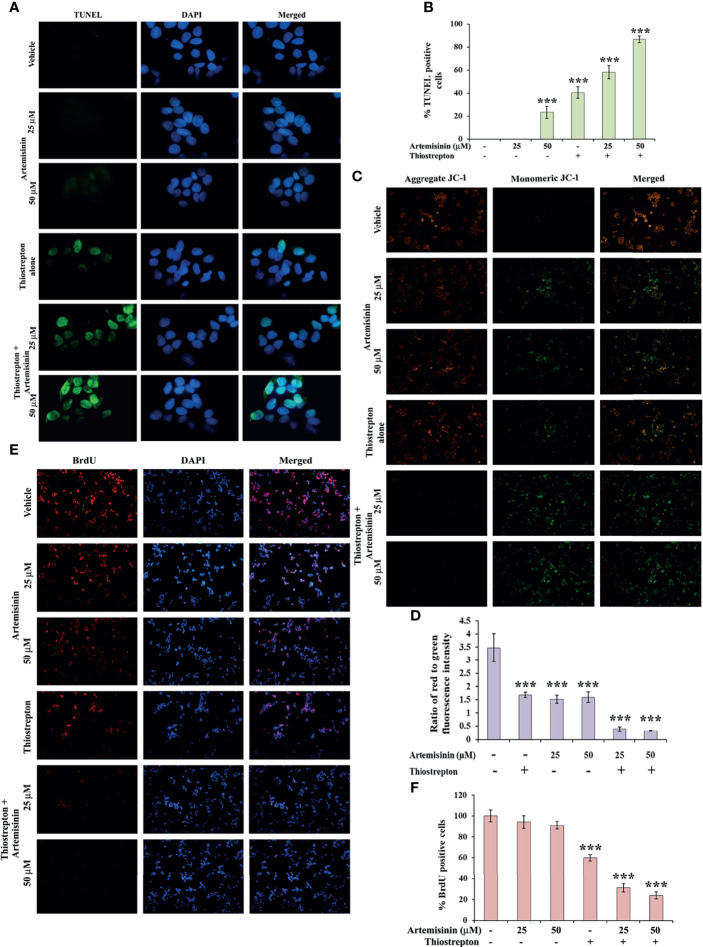
Artemisinin-induced cell death is accelerated by Thiostrepton. **(A)** Representative images (100X magnification) of TUNEL assay in HepG2 cells incubated with Artemisinin alone or in conjunction with Thiostrepton for 48 h are shown. **(B)** The percent apoptotic cells are graphically reported. **(C)** Representative images (40X magnification) of JC-1 assay of HepG2 cells treated with Artemisinin alone or in amalgamation with Thiostrepton for 48 h. **(D)** Graphical presentation of the ratio of measured red to green fluorescence intensity. **(E)** Representative images (40X magnification) of BrdU-positive HepG2 cells treated with either of the drugs alone or their combination for 48 h. **(F)** Graphical depiction of the percentage of BrdU positive cells. ***p < 0.001. All data are expressed as the means ± standard deviations from triplicate experiments. The two-tailed Student’s *t*-test was used to determine whether the differences between vehicle-treated set and Artemisinin-treated set were significant.

To explore the underlying cause behind the observed cell death (**Figure 5A**), we examined the levels of apoptotic markers. Western blotting revealed that Artemisinin, in consolidation with Thiostrepton, resulted in stronger cleavage of PARP, caspase 9 and caspase 7, compared to treatment with either agent (**Figure 5I**). In accordance, the population of apoptotic cancer cells as depicted by the TUNEL assay was found to be significantly augmented (60-85%) following co-administration with both drugs (**Figures 6A, B** **)**. Furthermore, apoptosis is typically characterized with deviations in the mitochondrial membrane permeability, alterations to which act as an early indication of apoptosis. Perturbation to the mitochondrial membrane was evaluated using JC-1 dye. While the vehicle treated HepG2 cells exhibited a high ratio of red to green fluorescence intensity, a significant decline (three-fold) in this ratio was observed following exposure to either Artemisinin or Thiostrepton (**Figures 6C, D** **)**, which was further reduced (seven-fold) in cells treated with the combination therapy, pointing towards a substantial effect of this drug cocktail on the mitochondrial membrane permeability.

Finally, BrdU assay was employed in an effort to understand the impact of this drug cocktail on the amount of active DNA synthesis as a reflection of proliferating cells in the S-phase of cell cycle. Treatment of HepG2 cells with the combination of Artemisinin and Thiostrepton yielded a clearly diminished population of BrdU-positive cells compared to single drug use (**Figures 6E, F** **)**, suggesting significant inhibition of cellular DNA synthesis of hepatic cancer cells. Our collated evidences, thus, highlight the promising anti-tumorigenic efficacy of Artemisinin combined with Thiostrepton.

### Thiostrepton Treatment of Resistant Hep3B Cells Sensitize Them to Artemisinin Treatment

**Figure 7:**
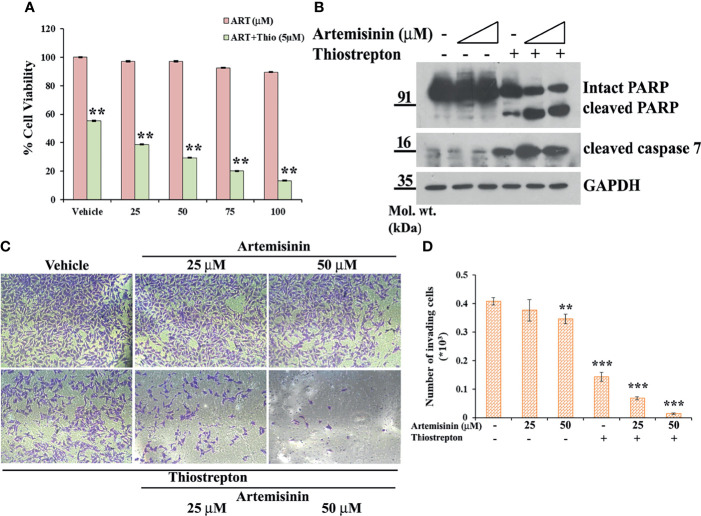
Resistant Hep3B cells respond to combined therapy of Thiostrepton and Artemisinin. **(A)** Hep3B cells were treated with indicated doses of Artemisinin either alone or in combination with Thiostrepton for 48 h, followed by assessment of cell viability using MTT assay. **(B)** Hep3B cells were treated with Artemisinin alone (25 and 50 µM) or in combination with Thiostrepton for 48 h. Western blot analysis was carried out using antibodies for cleaved PARP and cleaved caspase 7. GAPDH served as a loading control. **(C)** Representative images of invading cells after matrigel invasion assay performed in Hep3B cells following exposure to Artemisinin alone or in combination with Thiostrepton are provided. **(D)** Bar graph depicting the number of invading cells. **p < 0.01, and ***p < 0.001. All data are expressed as the means ± standard deviations from triplicate experiments. The two-tailed Student’s *t*-test was used to determine whether the differences between vehicle-treated set and Artemisinin-treated set were significant.

Our data has emphasized on the promising results of FoxM1 suppression in resistant HCC cells, such as the Hep3B cell line, in re-sensitizing them to anticancer therapeutics, including Artemisinin (**Figures 3**, **4**) and we found enhanced tumor suppressive impact of Artemisinin in presence of the FoxM1 inhibitor, Thiostrepton (**Figures 5**, **6**). This intrigued us to explore the possibility that exposing the resistant Hep3B cells to Thiostrepton may exert a sensitizing effect similar to what was obtained in case of FoxM1 knockdown. As anticipated, Artemisinin, on its own, failed to induce significant cytotoxicity in Hep3B cells. Importantly, when these cells were subjected to the combined treatment of Thiostrepton and Artemisinin, we found a substantial drop in Hep3B cell viability (**Figure 7A**). Furthermore, assessment of apoptotic markers showed distinct cleavage of PARP and caspase 7 in Hep3B cells, following exposure to Thiostrepton alone whereas the effect was considerably enhanced in the presence of the drug cocktail of Thiostrepton and Artemisinin (**Figure 7B**). Finally, invasion assay unearthed a remarkable (three-fold) decline in the invasive index of Hep3B cells in response to the treatment with Thiostrepton alone, suggesting the involvement of FoxM1 in driving neoplastic functions of HCC cells (**Figures 7C, D** **)**. Moreover, when used in conjunction with Thiostrepton, Artemisinin led to an eight-fold reduction in the number of invading cells (**Figures 7C, D** **)**. Therefore, taken together, our observations support the attractive potential of FoxM1 repression in re-sensitizing resistant HCC cells towards Artemisinin therapy.

## Discussion

Contemporary standard-of-care therapy for HCC includes pharmacotherapy, primarily sorafenib and regorafenib. Both the FDA approved drugs, however, exhibit limited clinical benefits due to drug resistance and off-target effects (3). Furthermore, developing novel hepatic cancer drugs is especially challenging for the risk for hepatotoxicity. Unsurprisingly, there is an urgent need for novel therapeutic modules for specific and efficient management of hepatic cancer. A plethora of published findings on the tumor inhibitory properties of Artemisinin and its derivatives in various human carcinomas, both in forms of mono- and combined therapy, has highlighted it to be profoundly safe and well-tolerated (65). We have demonstrated Artemisinin to cause a significant decline in different neoplastic properties of HCC cells (**Figure 1**), which is in agreement with published findings (22, 66). However, the underlying mechanisms of Artemisinin-mediated attenuation of tumor growth are still being uncovered. Till date, cell cycle arrest and apoptosis have been reported as the primary mode of action of Artemisinin and its derivatives in cancer cells with Bax, Bcl2, caspase 3, cyclinB1, Cdc2 and Mdm2 as its popularly recognized targets (67, 68).

Recently, FoxM1 silencing in head and neck carcinoma cells was found to augment cell cycle arrest induced by an Artemisinin derivative (47), hinting towards the prospective involvement of FoxM1 in the anticancer activities of Artemisinin. FoxM1 is a climacteric transcription factor, which stimulates cellular proliferation through transcriptional regulation of cell cycle-related genes (69). FoxM1’s strong connection to the known hallmarks of cancer in addition to its extensive and diverse role during tumorigenic development and progression have become apparent in the past decade, thus driving researchers to innovate FoxM1 inhibitors, including the thiazole antibiotics, siomycin A and thiostrepton (70), an ARF-peptide inhibitor (32) and the EGF receptor agonist, gefitinib (71). These studies lucidly indicate the importance of FoxM1 inhibition as a key to handicap tumorigenic advancements. We have demonstrated, for the first time, that Artemisinin-treated HCC cells displayed marked suppression of FoxM1 (**Figures 2A–H**) level and transcriptional activity (**Figures 2I–L** and **Supplementary Figure 3**), which culminates in decreased expression of its transcriptional targets, including Plk1, CyclinB1, Skp2 and Aurora B Kinase, among others. This rationalized, in part, the growth inhibitory properties of Artemisinin as such genes are involved directly in cell proliferation and cell cycle regulation (34). Dysregulated levels of mitotic drivers such as Plk1 and CyclinB1, Skp2, which typically contributes to timely degradation of key cell cycle drivers, and Aurora B Kinase that is involved in chromosomal segregation, spindle checkpoint assembly and cytokinesis, bestows tumor progression advantages (72–75). In harmony with such studies, data analysis of HCC patients further substantiated their role in hepatic carcinogenesis as we found that higher levels of these downstream targets of FoxM1 confer poor overall survival (**Supplementary Figure 1**). For instance, individuals with high expression of FoxM1 were associated with a mean survival of 25.2 months in comparison to patients displaying low expression of FoxM1, who demonstrated a mean survival of 61.7 months (**Supplementary Figure 1A**), further strengthening the potential role of FoxM1 and its targets in determining survival in HCC patients. Furthermore, it may be proposed that the decrease in FoxM1 mRNA level in the presence of Artemisinin may be due to Artemisinin-mediated inhibition of FoxM1 transactivation functions, which may result in blockage of the FoxM1 autoregulatory loop, consequently leading to decreased FoxM1 expression. However, this interesting area warrants further validation.

As re-iterated previously, a considerable clinical challenge in HCC treatment is imposed by drug resistance. Recognizing cellular factors that contribute towards this multifaceted phenomenon, therefore, pose an important sector of research to increase the death of therapy-resistant tumor cells. The presence of HBV, elevated EGFR activity and repressed AMPK activity in Hep3B cells have been attributed as the major determinants for the intrinsic resistance of these cells to sorafenib treatment (61). Furthermore, sorafenib-induced autophagy in HCC cells is an important tumor suppressive mechanism of this drug (62). However, lower autophagic responsiveness of Hep3B cells was shown to contribute to sorafenib resistance in Hep3B cells (61). On this note, sorafenib has been demonstrated to carry out its anticancer activities *via* FoxM1 inhibition in HCC cells (46). FoxM1 is intimately implicated in tumor recurrence of different malignancies (76, 77) while its down-regulation has been shown to sensitize resistant tumor cells to therapy (78). Of note, FoxM1 up-regulation directly correlates with sorafenib resistance in HCC models (46). In agreement, we found Hep3B cells to exhibit heightened level of FoxM1 compared to HepG2 cells (**Supplementary Figure 4**) and Artemisinin treatment in Hep3B cells could not stimulate significant downregulation of FoxM1 or its targets (**Figure 3C**). Our work, thus, strongly advocates that FoxM1 overexpression offers HCC cells a remarkable growth advantage (**Figures 3A, B** **)** and its targeted knockdown successfully sensitized conventionally resistant tumor cells to therapeutic regimes, including Artemisinin (**Figures 3D–G**, **4**). Deregulated expression of FoxM1 has been previously shown to promote heightened proliferation of HCC cells (41) and our observations suggest that FoxM1 knockdown augmented Artemisinin-related diminution of cell proliferation even in resistant HCC cells (**Figure 3E**). Consistent with the observation that FoxM1 silencing is connected to reduced tumorigenicity (79), we found Artemisinin to considerably mitigate growth and clonogenicity of Hep3BshFoxM1 cells, implying the potential of this natural product in delaying or preventing tumor relapse in cancer patients (**Figures 3F, G** **)**. Moreover, our data suggested strong inhibition of HCC cell migration and invasion (**Figure 4**) in Artemisinin-treated cells following FoxM1 silencing, foreboding the promising anti-metastatic prospect of Artemisinin. The observation further corroborates earlier studies that advocate FoxM1 deletion to drastically inhibit cancer cell invasion and metastasis (27, 32, 80, 81). In addition, activation of apoptotic signaling pathways is a key mechanism of cancer treatment modules, including chemo-, immuno-, radiation and gene therapy. Accordingly, the powerful ability of FoxM1 inhibition in resistant cancer cells to induce apoptosis in response to Artemisinin therapy points towards its encouraging therapeutic potential during cancer progression (**Supplementary Figure 5**).

Multiple studies have suggested that combination therapy imparts improved efficacy in comparison to monotherapy by augmenting the anticancer activity, minimizing adverse effects and reducing the risk of resistance. A huge spectrum of agents can be potentially combined with Artemisinin and its derivatives (82). Given our novel observation of re-sensitization of resistant HCC cells to Artemisinin therapy following FoxM1 depletion, the efficacy of contemporary chemotherapeutic regimes is expected to improve in presence of FoxM1 inhibitors. In the present study, we found that even at low dosage, Artemisinin, when combined with Thiostrepton, acted in an additive manner and resulted in enhanced suppression of FoxM1 level in HepG2 cells, compared to treatment with either of the drugs alone (**Supplementary Figure 6**). The combinatorial regime further manifested significantly greater inhibition on the growth, migration, invasion, anchorage-independent colony formation and produced considerably enhanced mitochondrial membrane damage and apoptotic cell death than either agent applied alone (**Figures 5** **–** **7**) and this was true for both sensitive (**Figures 5**, **6**) as well as resistant (**Figure 7**) HCC cells, evoking the usefulness of such novel combination therapy, particularly for treatment of late stage liver tumors to improve the mortality and morbidity of such patients. Thus, rational amalgamation of anticancer drugs can exert more robust antitumor effects with reduced cytotoxicity to normal cells.

**Figure 8:**
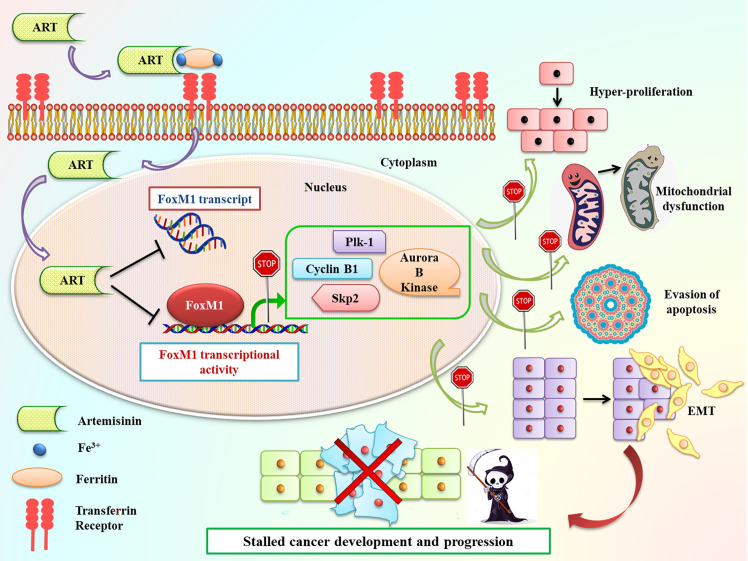
Schematic representation of Artemisinin-mediated anticancer effects through FoxM1 repression. Following incubation of cancer cells with Artemisinin, it is taken up by the transferrin receptors present on the cell surface along with iron-loaded ferritin and transported inside the cell. Once inside the cell, we found that Artemisinin treatment transcriptionally repressed FoxM1 mRNA level and disrupted its *trans*-activation. The regulated expression of many of the transcriptional targets of FoxM1, such Plk1, cyclinB1, Skp2 and Aurora B kinase is necessary to prevent cell cycle deregulation, a primary hallmark of cancer. Aberrant FoxM1 transcriptional activity leads to untimely expression of these target genes, which is a common occurrence in cancers and, thereby, promotes neoplastic transformations. Artemisinin therapy blocked this perturbed transcriptional activation of FoxM1 downstream targets and led to downregulation in their expression, thus hampering various cancer hallmarks, ultimately culminating in reduced carcinogenesis.

In summary, our current findings exhibited that Artemisinin commendably repressed FoxM1 at its transcriptional level and curbed its downstream *trans*-activation ability, thus delaying cancer cell proliferation, relegating tumor growth and increasing apoptosis (**Figure 8**). Due to their need for rapid proliferation and growth, cancer cells have a voracious appetite for enhanced iron uptake- to facilitate this, cancer cells have, on their surface, transferrin receptors in much greater number than normal cells. These transferrin receptors allow the entry of Artemisinin into cancer cells (20). The differential iron preference allows cancer cells to favorably take up Artemisinin, thus explaining, in part, the minimal non-specific toxic effects associated with this phytochemical. Our observations showed that, once inside the cancer cells, Artemisinin-mediated blockage of FoxM1 *trans*-activation results in downregulation of major transcriptional targets of FoxM1, such as Plk1, cyclinB1, Skp2 and Aurora B kinase. We have already emphasized that overexpression of such genes have been shown to confer tumorigenesis. Therefore, Artemisinin-induced diminution in their levels is expected to repress carcinogenic progression (**Figure 8**). We also demonstrated the superior therapeutic efficacy of Artemisinin in collocation with FoxM1 suppression either through its knockdown or *via* its inhibition by Thiostrepton in HCC model. A number of clinical trials are ongoing for the usage of Artemisinin derivatives in colorectal, cervical, metastatic breast and lung malignancies, thus illuminating Artemisinin as a feasible treatment option for cancer patients (83). Controlled and randomized clinical trials need to be underway to assess both the efficacy and tolerability of our combinatorial approach between Artemisinin and Thiostrepton for efficient cancer therapy for broad use in various types of malignancies.

## Data Availability Statement

The original contributions presented in the study are included in the article/**Supplementary Material**. Further inquiries can be directed to the corresponding author.

## Author Contributions

DN: conception and design, development of methodology, data acquisition, analysis and interpretation, manuscript writing. PC: data collection and analysis, manuscript editing. AS: data collection and manuscript editing. HB: data calculations, statistical analysis and manuscript editing. AN: conception and design, development of methodology, data analysis and interpretation, overall supervision, manuscript writing and polishing. All authors contributed to the article and approved the submitted version.

## Funding

This work was supported by research grants to AN funded by Council of Scientific and Industrial Research (CSIR no. 37 (1682)/17/EMR-II), Department of Biotechnology (DBT no.BT/PR15422/MED/30/1705/2016), DST-SERB (CRG/2020/003380), Faculty Research Program Grant – Institute of Eminence of Delhi University (IoE/FRP/LS/2020/27 and IoE/2021/12/FRP) and University Grants Commission (UGC-SAP Program). The funders had no role in the experimental designs or data collection and analysis or the decision to submit the work for publication.

## Conflict of Interest

The authors declare that the research was conducted in the absence of any commercial or financial relationships that could be construed as a potential conflict of interest.

## Publisher’s Note

All claims expressed in this article are solely those of the authors and do not necessarily represent those of their affiliated organizations, or those of the publisher, the editors and the reviewers. Any product that may be evaluated in this article, or claim that may be made by its manufacturer, is not guaranteed or endorsed by the publisher.
