# Supplementary material for: Artemisinin Mediates Its Tumor-Suppressive Activity in Hepatocellular Carcinoma Through Targeted Inhibition of FoxM1
Source: Front Oncol. 2021 Nov 24;11:751271. doi: 10.3389/fonc.2021.751271 (PMC8652299; doi:10.3389/fonc.2021.751271)
Supplement: Supplementary file 1 [file DataSheet_1.docx]

**Supplementary Figures**

**
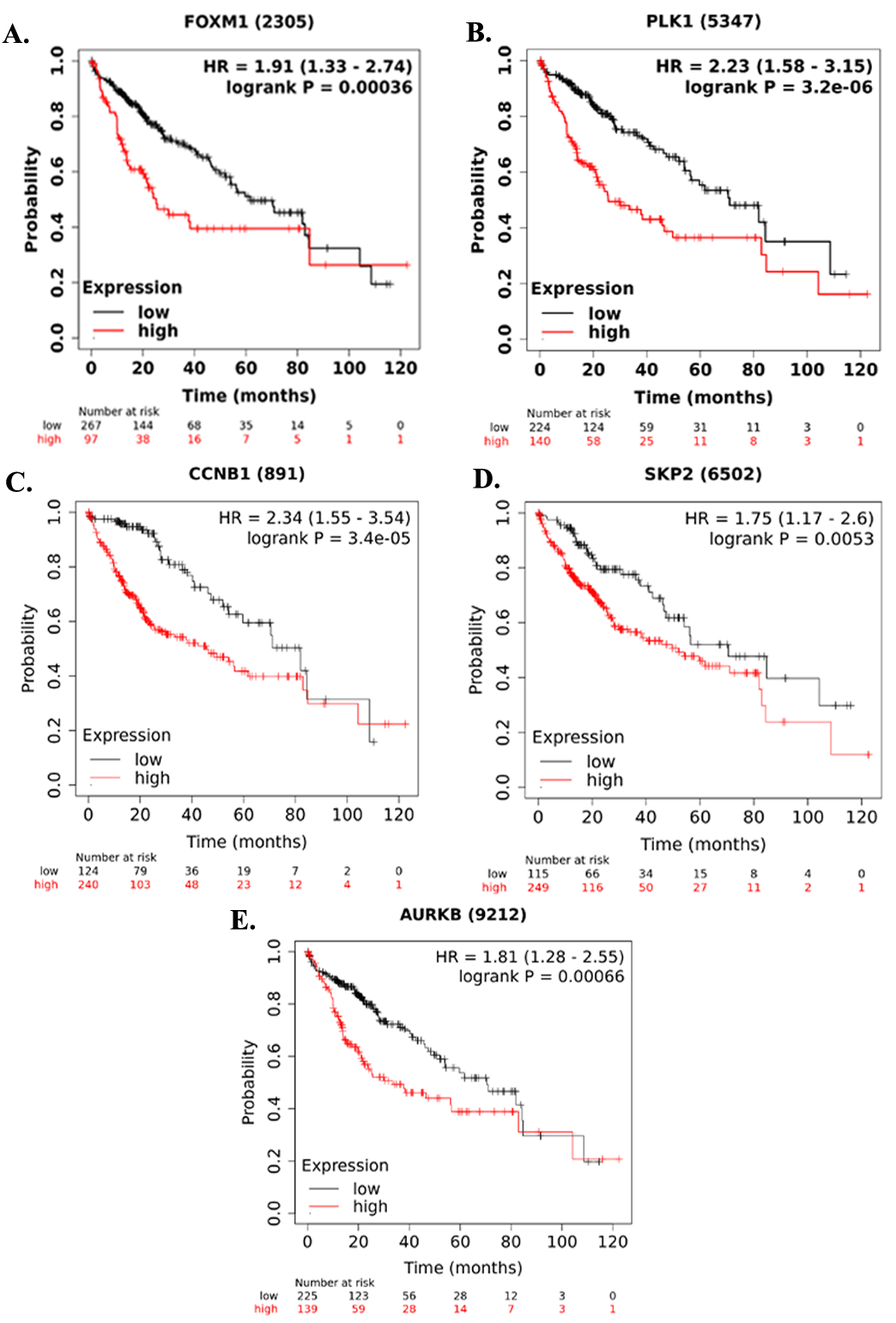
**

**Supplementary Figure 1. Overexpression of FoxM1 and its notable targets correlates with lower overallsurvival in HCC patients.** Representative Kaplan-Meier plots of HCC patients showing overall survival in high and low expressing cohorts for **(A)** FoxM1, **(B)** Plk1, **(C)** CyclinB1 (CCNB1), **(D)** Skp2 and **(E)** Aurora B Kinase (ABK) were analysed using dataset available in cBioportal. No restriction was incorporated for staging, grade, invasive nature, gender, race, sorafenib treatment or alcohol consumption for our analysis.


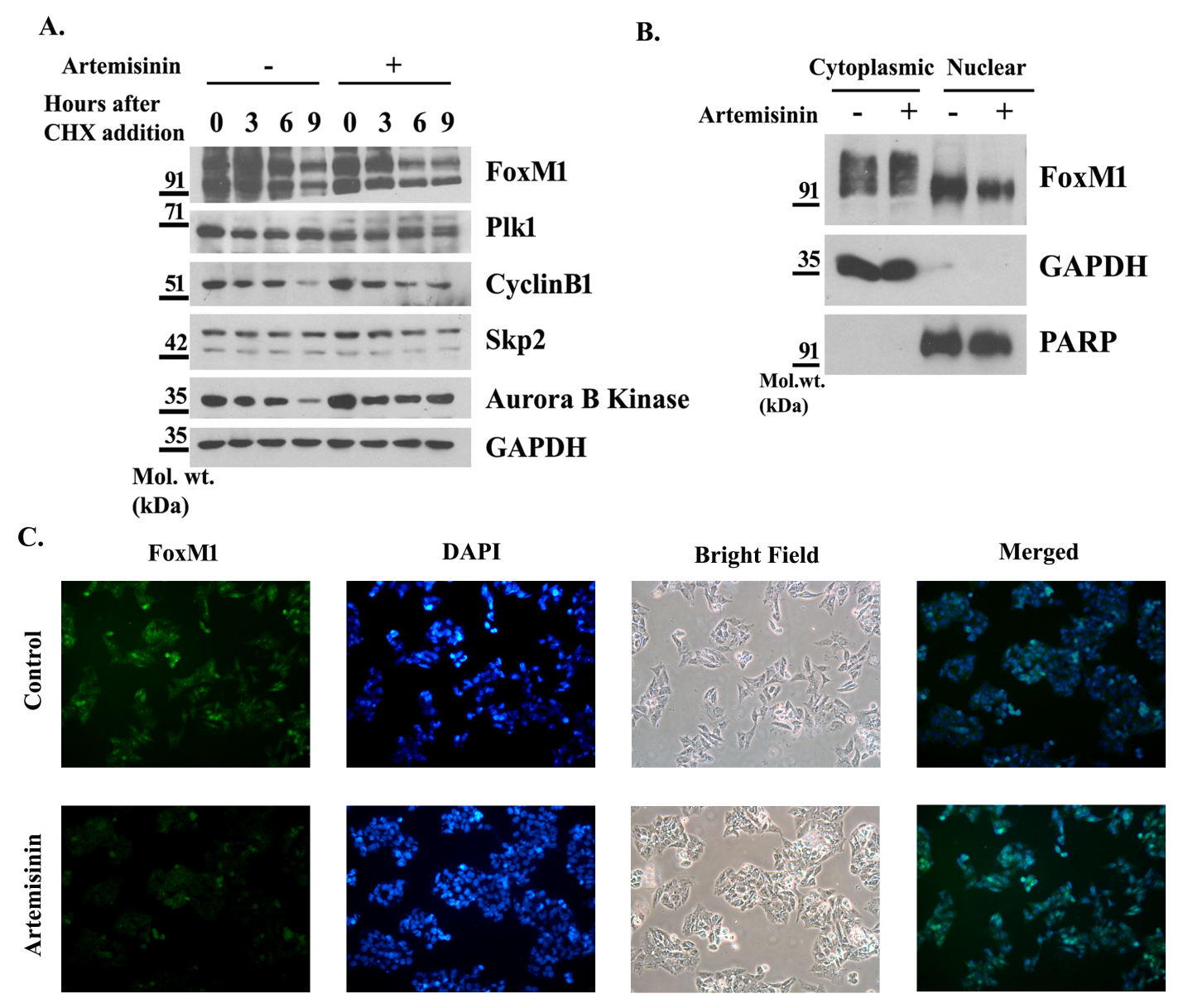


**Supplementary Figure 2. FoxM1 protein turnover and cellular distribution are unaffected by Artemisinin.** **(A)** HepG2 cells were treated with 100 µM Artemisinin for 48 h followed by addition of cycloheximide (100 µg/ml). Cells were harvested at indicated time-points and subjected to SDS-PAGE and western blotting with antibodies specific for FoxM1, Plk1, CyclinB1, Skp2 and Aurora B Kinase. GAPDH was used as a loading control. **(B)** HepG2 cells treated with 100 µM Artemisinin for 48 h were subjected to nuclear cytoplasmic fractionation. GAPDH was used as a cytoplasmic marker and PARP served as a nuclear marker. **(C)** HepG2 cells were exposed to 100 µM Artemisinin for 48 h. Cells were immunostained with anti-FoxM1 antibody, followed by detection with Alexa Fluor 488-tagged secondary antibody. Nuclei were counterstained with DAPI. Representative image (40X magnification) from fluorescence microscope has been presented here. Data is representative of at least three independent experiments.


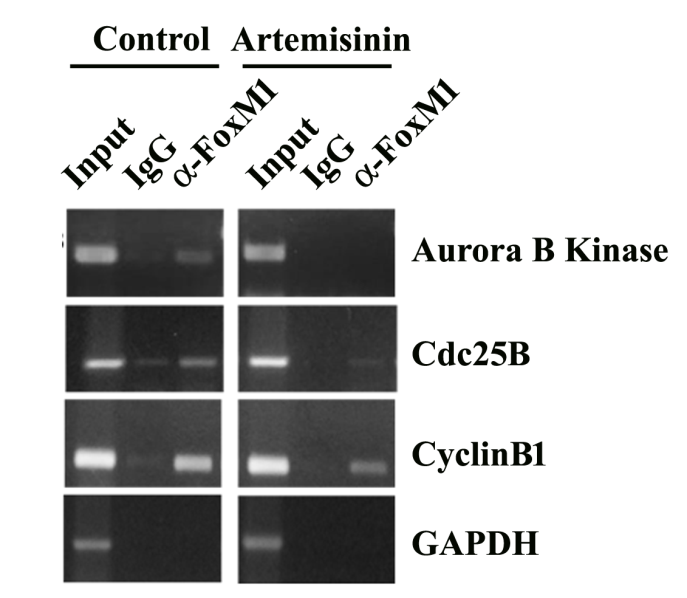


**Supplementary Figure 3. Artemisinin disrupts FoxM1 *trans*-activation ability.** HepG2 cells treated with either vehicle or 100 µM Artemisinin for 24 h were subjected to ChIP using anti-FoxM1 antibody followed by PCR amplification with primers against Aurora B Kinase, Cdc25B and CyclinB1. GAPDH served as an internal control. Representative ChIP image from at least three independent experiments is shown.





**Supplementary Figure 4. Comparative analysis of FoxM1 level in HCC cells.** Equal amounts of whole cell lysates from Hep3BpSuper and Hep3BshFoxM1 stable cell lines in addition to HepG2 cells were subjected to SDS-PAGE and immunoblotting with anti-FoxM1 antibody. GAPDH was used as a loading control. Representative image from at least two independent experiments is shown.

**
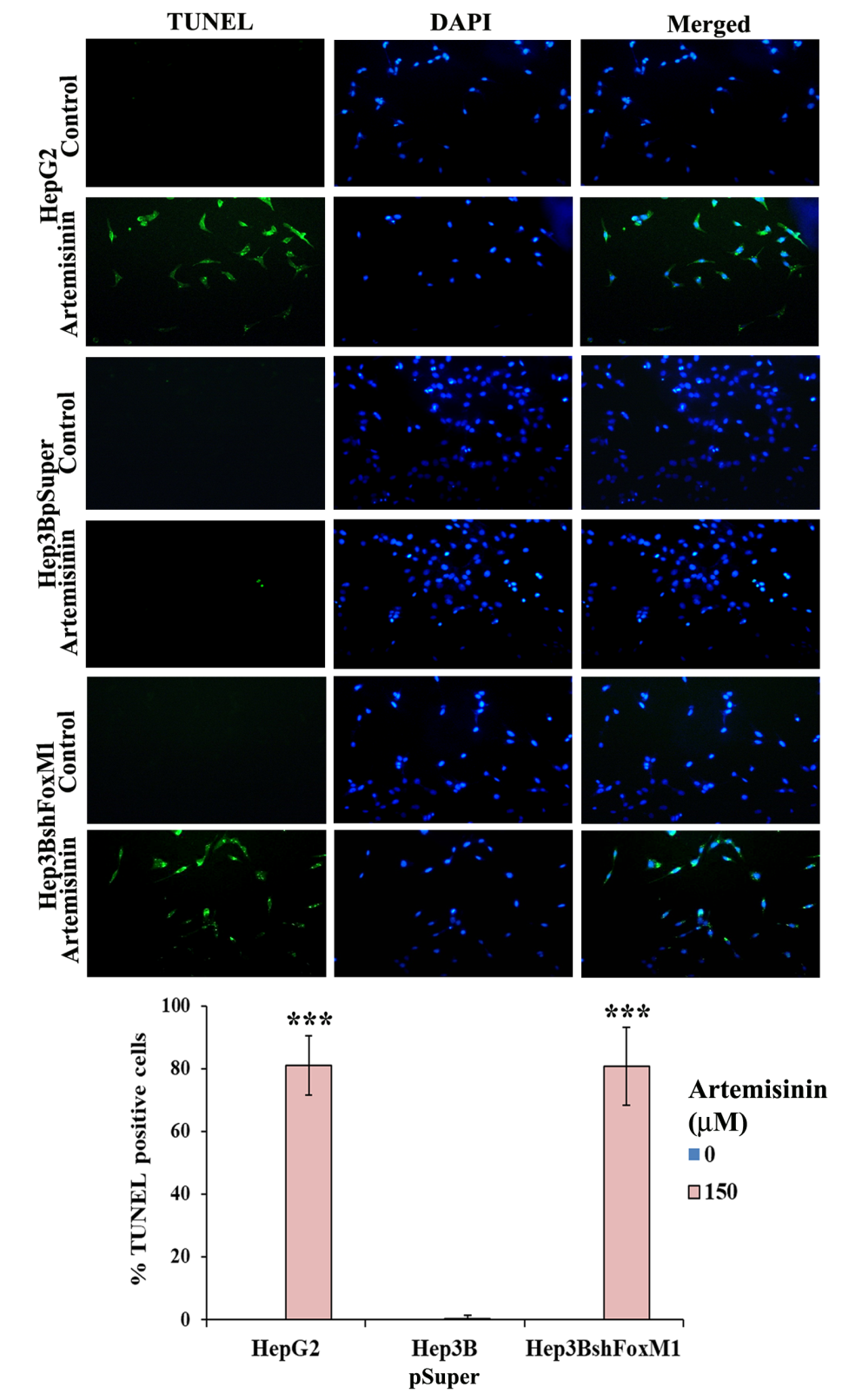
**

**Supplementary Figure 5. FoxM1 knockdown induces apoptosis in Artemisinin- treated Hep3B cells.** Apoptotic effect was determined using TUNEL assay. HepG2, Hep3BpSuper and Hep3BshFoxM1 cells were treated with either vehicle or 150 µM Artemisinin for 24 h and subjected to TUNEL assay as described in Materials and Methods. Representative image depicts the TUNEL-positive cells (left panel) and their merged version with DAPI (right-most panel). *** *p* < 0.001. All data are expressed as the means ± standard deviations from triplicate experiments. The two-tailed Student's *t*-test was used to determine whether the differences between vehicle-treated set and Artemisinin-treated set were significant.





**Supplementary Figure 6. Combined treatment with Artemisinin and Thiostrepton appreciably suppressed FoxM1 level in HepG2 cells. (A)** HepG2 cells were treated with either vehicle or 1.5, 3 and 5 µM Thiostrepton for 48 h. Equal amount of whole cell lysates were subjected to SDS-PAGE, followed by western blotting with anti-FoxM1 antibody. GAPDH served as a loading control. **(B)** HepG2 cells were treated with Artemisinin (25 and 50 µM), either alone or in combination with 5 µM Thiostrepton for 48 h. Equal amounts of whole cell lysates were subjected to SDS-PAGE, followed by western blotting with anti-FoxM1 antibody. GAPDH served as a loading control. Representative image from at least two independent experiments is provided.
